# Concomitant Activation of *OsNAS2* and *OsNAS3* Contributes to the Enhanced Accumulation of Iron and Zinc in Rice

**DOI:** 10.3390/ijms24076568

**Published:** 2023-03-31

**Authors:** Sichul Lee, Md Mizanor Rahman, Hiromi Nakanishi, Naoko K. Nishizawa, Gynheung An, Hong Gil Nam, Jong-Seong Jeon

**Affiliations:** 1 Center for Plant Aging Research, Institute for Basic Science (IBS), Daegu 42988, Republic of Korea; 2 Department of Agricultural Biotechnology, National Institute of Agricultural Science, Jeonju 54896, Republic of Korea; 3 Graduate School of Green-Bio Science and Crop Biotech Institute, Kyung Hee University, Yongin 17104, Republic of Korea; 4 Department of Global Agricultural Sciences, Graduate School of Agricultural and Life Sciences, The University of Tokyo, Tokyo 113-8655, Japan; 5 Research Institute for Bioresources and Biotechnology, Ishikawa Prefectural University, Nonoichi 921-8836, Japan; 6 Department of New Biology, DGIST, Daegu 42988, Republic of Korea

**Keywords:** iron, zinc, activation mutant, nicotianamine synthase, biofortification, rice

## Abstract

Nicotianamine (NA) is produced by NA synthase (NAS), which contains three genes in rice and is responsible for chelating metals such as iron (Fe) and zinc (Zn), as well as preserving metal homeostasis. In this study, we generated a transgenic plant (*23D*) that shows simultaneous activation of *OsNAS2* and *OsNAS3* by crossing two previously identified activation-tagged mutants, *OsNAS2-D1* (*2D*) and *OsNAS3-D1* (*3D*). Concomitant activation of both genes resulted in the highest Fe and Zn concentrations in shoots and roots of the *23D* plants grown under normal conditions and Fe and Zn limited growth conditions. Expression of genes for the biosynthesis of mugineic acid family phytosiderophores (MAs) and Fe and Zn uptake were enhanced in *23D* roots. Additionally, *23D* plants displayed superior growth to other plants at higher pH levels. Importantly, *23D* seeds had NA and 2′-deoxymugineic acid (DMA) concentrations that were 50.6- and 10.0-fold higher than those of the WT. As a result, the mature grain Fe and Zn concentrations of the *23D* plant were 4.0 and 3.5 times greater, respectively, than those of the WT. Furthermore, *23D* plants exhibited the greatest resistance to excess metals. Our research suggests that simultaneous activation of *OsNAS2* and *OsNAS3* can enhance Fe and Zn accumulation in rice grains while also increasing plant tolerance to growing situations with metal deficiency and excess metal availability.

## 1. Introduction

For proper growth and development of plants and humans, iron (Fe) and zinc (Zn) are essential micronutrients and accumulate in edible parts of crops that are primary sources of micronutrients for humans [1]. In plants, Fe is required for electron transfer reactions in mitochondria, chlorophyll biosynthesis, and photosynthesis [2,3]. Zn is a structurally important component for almost 10% of all plant proteins; affects protein–protein interactions in plant cells; and is required as a cofactor for the functional activity of various enzymes such as Cu/Zn superoxide dismutases, carbonic anhydrase, and alkaline phosphatase [4,5]. In the human body, Fe takes part in various cellular functions, such as immunity, oxygen transport, cellular respiration, energy metabolism, electron transfer reactions, and cell division and differentiation [6,7]. Zn plays a critical role as an essential component for over 300 enzymes and 2000 transcription factors, where any alteration in its quantity can affect signaling and enzymatic responses intracellularly [8].

Fe and Zn content in rice grains is low, as in other cereal crops such as maize and wheat, and populations that depend on these grains as a staple food experience hidden hunger [2,9,10]. As a result, the prevalence of Fe deficiency anemia (IDA) is believed to be above 33% worldwide, while up to 20% may have zinc deficiency [2]. Biofortification, which uses several strategies such as agronomic practices, traditional plant breeding, and modern biotechnology, is a practical and sustainable method to generate nutritionally enhanced food crops with increased bioavailability to the human population [11]. Transgenic approaches are being used for increasing Fe and Zn concentrations in rice grains, utilizing different genes under the control of constitutive or tissue-specific promoters, either a single gene alone or multiple genes together, which are involved in different regulatory pathways including metal-homeostasis-related genes, metal transporters, the MA pathway, and others [2].

Nicotianamine (NA), a non-proteinogenic amino acid that chelates many metal cations, including Fe, Zn, copper (Cu), and manganese (Mn), is found in all plants examined and plays an important role in the internal transport of the metals to seeds [12]. In addition, it serves as a precursor of phytosiderophore 2′-deoxymugineic acid (DMA), a Fe (III) chelator secreted from rice roots [13]. Thus, it was suggested that enhanced NA could possibly translocate more Fe and Zn into seeds in rice [13]. Three molecules of S-adenosyl methionine are converted into NA by NA synthase (NAS) [14]. In the rice genome, three *NAS* genes are present, namely, *OsNAS1*, *OsNAS2*, and *OsNAS3*, which are differentially regulated by the availability of the external Fe status [15].

Overexpression of *NAS* genes in rice resulted in elevated concentrations of NA, DMA, Fe, Zn, and other micronutrients in rice grains with enhanced Fe and Zn bioavailability, and moreover, plants were more resistant to Fe and Zn deficiencies and excess metal toxicity [16,17,18,19,20,21]. For example, increased expression of *OsNAS3* in an activation-tagged mutant resulted in enhanced concentrations of Fe (2.9-fold), Zn (2.2-fold), and Cu (1.7-fold) in its grains than wild type (WT). Moreover, NA and DMA levels were also increased, 9.6-fold and 4.0-fold, respectively [17]. Enhanced expression of *OsNAS2* in an activation-tagged mutant and maize *ubiquitin*-promoter-driven *OsNAS2* transgenic plants produced seeds with 2.3- to 3.0-fold more Fe, and 2.3- to 3.2-fold more Zn, along with 19.8-fold more NA and 3.5-fold more DMA than the WT seeds [18,19]. When all three rice *OsNAS* genes were overexpressed using *CaMV35S* promoter, unpolished grains of transgenic seeds contained 2.4, 3.5, and 2.7 times more Fe, respectively, in comparison to WT, while Zn concentrations were increased by 1.9, 2.5, and 2.1 times, respectively [16,22]. There was also a significant increase in NA content. Overexpression of *OsNAS1* under the control of the endosperm-specific promoter *OsGluB1* (*pGluB1)* produced transgenic grains with higher NA content than WT that resulted in higher Fe and Zn concentrations in brown rice, and they were more Fe bioavailable, even after the grains were polished [23]. Transgenic rice plants carrying the barley *NAS* gene, *HvNAS1,* under the control of *pGluB1* or rice *actin1* promoter produced seeds with Fe levels that were 1.5 times or 1.3 times higher, respectively [20,21].

Transgenic rice plants coexpressing *NAS* with other genes are being generated to further improve Fe and Zn concentrations in rice grains [2]. Fe concentration increased over sixfold in rice endosperm when coexpressing the *Ferritin* gene from *Phaseolus vulgaris* (*PvFer*) and the *Arabidopsis thaliana NAS* gene (*AtNAS1*) [24]. Coexpression of *OsNAS1* and barley *nicotianamine aminotransferase* (*HvNAATb*) genes under maize *ubiquitin* promoter resulted in a 1.4- to 3.7-fold increase in Fe and 1.2- to 4.2-fold increase in Zn in the rice endosperm [25,26]. Trijatmiko et al. [27] used a single cassette containing *OsNAS2* driven by the *CaMV35S* promoter and the soybean *ferritin* gene (*GmFer-H1*) under the control of the endosperm-specific promoter *GlutelinA2* (*pGluA2*) to generate transgenic plants in the IR64 variety, and they found 7.5-fold higher Fe and 2.7- to 3.8-fold higher Zn concentrations in polished grains compared with WT. When several genes were introduced into rice, including a construct containing *AtIRT1* that encodes an Iron-Regulated Transporter1, that was driven by its native promoter and another construct containing *PvFer* that was driven by *OsGlB-1* promoter and *AtNAS1* driven by *CaMV35S* promoters, concentrations of Fe and Zn were increased up to 3.8-fold and 1.8-fold, respectively, in polished grains compared to the WT [28]. When a single construct containing the tissue-specific expression of the *ZmYS1* transporter driven by the *OsHMA2* promoter and the *TOM1* transporters by *OsFRDL1* was combined with endosperm-specific expression of *ferritin* and the constitutive expression of *NAS*, Fe and Zn concentrations were increased up to 9.3- fold and 1.2-fold, respectively, compared with WT [29]. However, none of the studies that employed the gene stacking method included multiple *OsNAS* genes.

In this study, we generated a transgenic rice line (*23D*) with simultaneous activation of *OsNAS2* and *OsNAS3* by crossing two rice activation-tagged mutants, *OsNAS2-D* (*2D*) and *OsNAS3-D* (*3D*) [17,18,19]. Mature grains of *23D* contained increased levels of NA, DMA, Fe, and Zn compared to the *2D*, *3D*, and WT. In addition, we showed that *23D* plants exhibited the highest tolerance to Fe and Zn deficiencies and excess metal toxicities in comparison with the *2D*, *3D*, and WT plants.

## 2. Results

### 2.1. Isolation of Double-Activation-Tagged Mutant and Its Enhanced Tolerance to Fe and Zn Deficiencies

Previously, two activation-tagged mutants of rice *NAS* genes, *OsNAS2-D1* and *OsNAS3-D1,* were studied to increase micronutrient content, specifically Fe and Zn [17,18,19]. Because simultaneous manipulation of multiple genes to increase micronutrient content is a good practice to enhance the nutritive value of grains, we attempted to increase *OsNAS2* and *OsNAS3* expressions together. For this aim, we crossed the two activation-tagged mutants, *OsNAS2-D1* (*2D*) and *OsNAS3-D1* (*3D*), and isolated a transgenic line containing both activation-tagged transgenes (*23D*) by PCR. Then, we analyzed the expressions of *OsNAS2* and *OsNAS3* in seedling shoots and roots, and flag leaves of *2D*, *3D,* and *23D* along with the WT (Figure S1). Compared to WT, significant expression increases of both genes were observed in all tissues, shoots, and roots of seedlings and flag leaves, tested of *23D*, whereas *OsNAS3* expression did not alter in *2D* and *OsNAS2* expression did not alter in *3D* (Figure S1).

**Figure 1:**
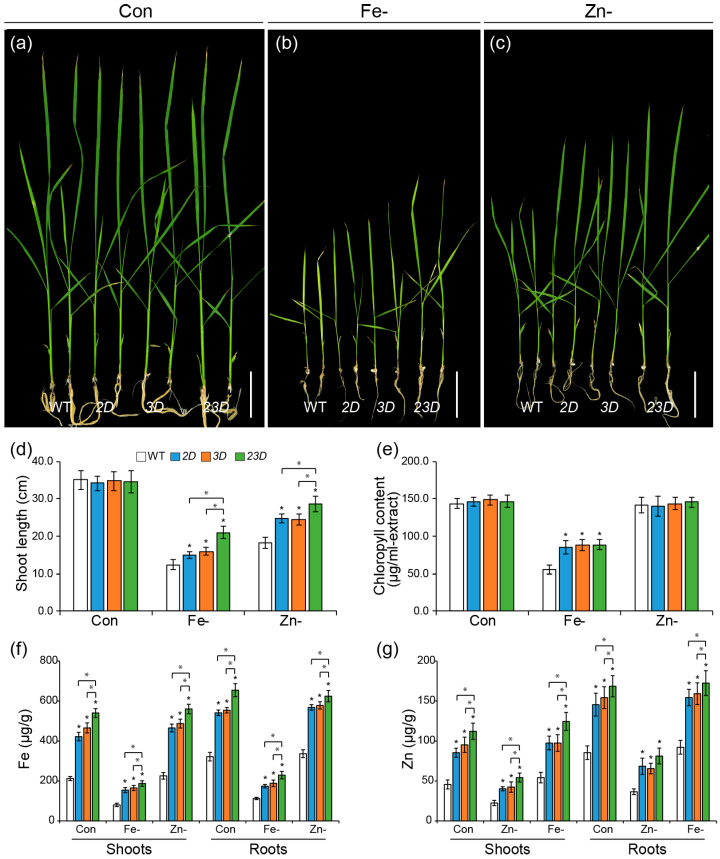
Tolerance of WT and activation-tagged mutants to Fe and Zn deficiencies. (**a**–**c**) Phenotypes of WT, *OsNAS2* activating-tagged mutant (*2D*), *OsNAS3* activating-tagged mutant (*3D*), and double activation-tagged mutant (*23D*) grown under control Yoshida solution (Con), Fe-deficient (Fe-) solution or Zn-deficient (Zn-) solution for 15 days. Bars = 5 cm. Shoot lengths (**d**), total chlorophyll concentrations (**e**), Fe concentrations (**f**), and Zn concentrations (**g**) in shoots and roots of WT and activation-tagged mutants. Data are mean ± SE (n = 10 for length; n = 4 for measurements of chlorophyll contents and Fe and Zn concentrations in shoots and roots). Significant differences among WT and mutant plants are indicated with an asterisk (* *p* < 0.05 by Student’s *t*-test).

We raised the mutant seedlings in Fe- and Zn-deficient medium to examine whether the elevated expression of *OsNAS2* and/or *OsNAS3* could have any effect on growth and metal distribution (Figure 1 and Figure S2). Seedlings of all mutants did not show any significant change in shoot length and chlorophyll content on the control media, Yoshida solution, and a half-strength MS medium (Figure 1a,d,e and Figure S2a,d,e). However, significant differences were observed in all mutant seedlings grown under Fe- and Zn-deficient media compared to those of the WT (Figure 1b–e and Figure S2b–e). *23D* seedlings outperformed all other seedlings, with lengths of 69.4 and 56.4% higher than the WT seedlings on Fe- and Zn-deficient media, respectively, while the lengths of *2D* seedlings were 21 and 35.6% more, and *3D* seedlings were 28.6 and 34.2% more on Fe and Zn deficient media, respectively, compared to the WT seedlings. Moreover, all transgenic seedlings had less chlorosis when Fe was limited, as compared to the WT (Figure 1e and Figure S2e). For example, chlorophyll concentrations for *23D*, *2D*, and *3D* were increased to 159.4%, 153.8%, and 157.6%, respectively, compared to the WT.

To evaluate whether simultaneous activation of *OsNAS2* and OsNAS3 affected metal distribution, we measured Fe and Zn levels in shoots and roots of the mutants and WT seedlings (Figure 1f,g). We found that both metals were increased significantly in the mutants, irrespective of external metal supply, compared to that of the WT (Figure 1f,g). It is noteworthy that *23D* seedlings had the highest Fe and Zn contents, compared with those of *2D*, *3D*, and WT (Figure 1f,g). Under the control growth conditions, the Fe content in the shoots of *2D*, *3D*, and *23D* seedlings was increased to 198.6%, 218.9%, and 254.2%, respectively, compared with WT, while the corresponding root levels increased to 168.3%, 172.4%, and 203.5%, respectively (Figure 1f). Under Fe deficiency, Fe content in shoots of *2D*, *3D*, and *23D* seedlings increased to 191.5%, 205.2%, and 232.9%, respectively, compared with WT, whereas the corresponding root values rose to respective values of 154.8%, 167.5%, and 203.7% (Figure 1f). Under Zn deficiency, Fe concentration in shoots and roots of *23D* seedlings were highest out of the plants (Figure 1f). That is, the Fe contents in the shoots of *2D*, *3D*, and *23D* seedlings were increased to 206.3%, 216.1%, and 232.9%, respectively, compared with WT, while the corresponding root levels increased to 168.9%, 171.8%, and 203.7%, respectively (Figure 1f).

In addition, compared to WT, Zn concentrations in the shoots of *2D*, *3D*, and *23D* seedlings grown on Zn sufficient medium increased by 187.1%, 208.6%, and 245.8%, respectively, while the corresponding root values increased by 170.3%, 180.5%, and 197.2%, respectively (Figure 1g). Zn contents of *2D*, *3D*, and *23D* seedling shoots increased under Zn deficiency to 179.3%, 188.6%, and 241.6%, respectively, compared to WT, whereas the corresponding root values increased to 187.3%, 179.4%, and 222.4%, respectively (Figure 1g). Even under Fe deficiency, Zn concentrations in shoots and roots of *23D* seedlings were higher than that of other plants same as normal and Zn deficiency conditions (Figure 1g).

**Figure 2:**
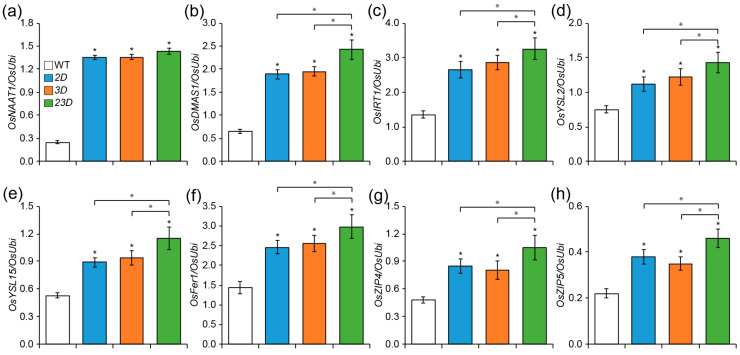
Quantitative RT-PCR analysis of Fe and Zn homeostasis-related genes at the seedling roots. Plants were grown under the control Yoshida solution for 10 days. Levels of *OsNAAT1* (**a**), *OsDMAS1* (**b**), *OsIRT1* (**c**), *OsYSL2* (**d**), *OsYSL15* (**e**), *OsFer1* (**f**), *OsZIP4* (**g**), and *OsZIP5* (**h**) in the roots of WT and activation-tagged mutants. Significant differences among WT and mutant plants are indicated with an asterisk (* *p* < 0.05 by Student’s *t*-test).

The above data prompted us to investigate the transcript abundance of various genes involved in the homeostasis of Fe and Zn (Figure 2): the *nicotianamine aminotransferase* gene *OsNAAT1* and *2′-deoxymugineic acid synthase* gene *OsDMAS1,* which act downstream of *OsNAS* genes and are involved in the synthesis of MAs [30,31]; *OsIRT1* and *OsYSL15* (*Yellow Stripe1-Like 15*), which uptake Fe from the soil as membrane transporters [32,33,34,35]; *OsYSL2*, which acts as NA-metal transporter and facilitates Fe translocation [36,37]; rice *ferritin1* (*OsFer1*), which acts as a principal Fe storage protein [38]; and *OsZIP4* and *OsZIP5*, which encode a membrane protein that transports Zn [39,40]. Transcript levels of these genes were found to be significantly higher in the roots of all the mutants compared to the WT (Figure 2). Overall, we found that the roots of *23D* had the highest level of all the transcripts we looked at in comparison to others (Figure 2).

### 2.2. Expression Analysis of Three OsNAS Genes and the Comparison of Seedling Growth under High pH Conditions

**Figure 3:**
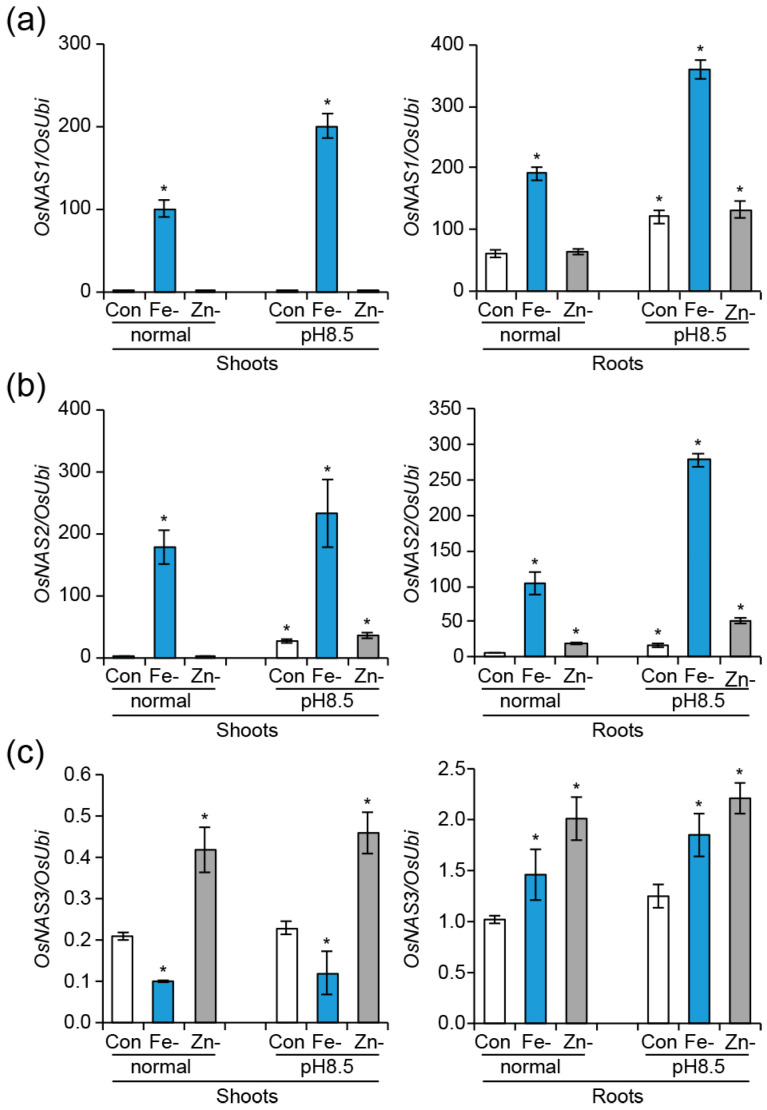
Elevated expression of *OsNAS* genes by high pH. Expression of *OsNAS1* (**a**), *OsNAS2* (**b**), and *OsNAS3* (**c**) grown under normal (pH 5.5) or high pH (pH 8.5) Yoshida solution with Fe and Zn (Con), or without Fe (Fe-) or Zn (Zn-). Plants were grown in normal Yoshida solution for 7 days and thereafter were transferred to each growth condition. The seedlings were grown for an additional three days, and shoots and roots from the seedlings were sampled for RNA isolation. Significant differences between the control (pH 5.5) and each growth condition are indicated with an asterisk (* *p* < 0.05 by Student’s *t*-test).

**Figure 4:**
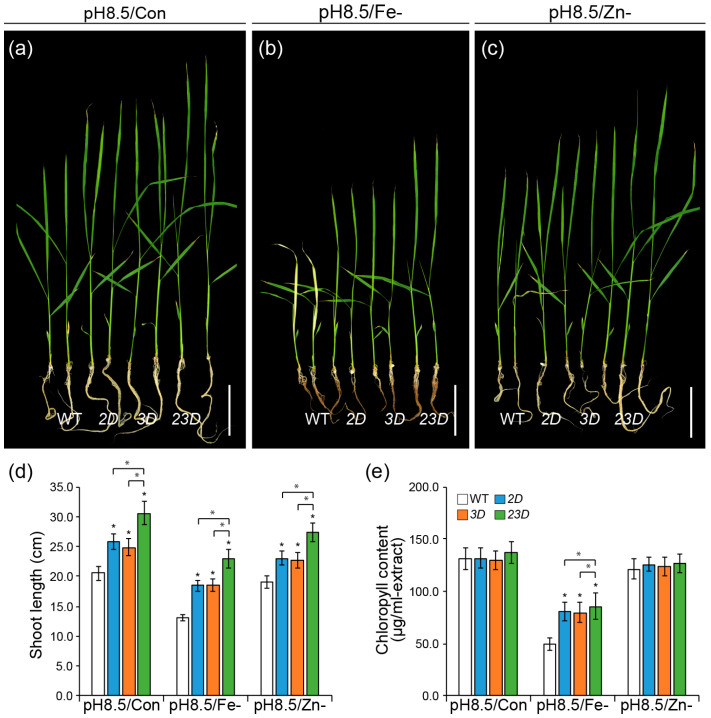
Comparison of seedling growth test under high pH conditions. (**a**–**c**) Phenotypes of WT, *2D*, *3D*, and *23D* grown on alkaline Yoshida solution ((**a**) pH 8.5), without Fe ((**b**) p H8.5/Fe-), or without Zn ((**c**) p H8.5/Zn-) for 15 days. Bars = 5 cm. Shoot lengths (**d**) and total chlorophyll concentrations (**e**) of WT and activation-tagged mutants. Data are mean ± SE (n = 8 for shoot length; n = 4 for chlorophyll content), and significant differences among mutants and WT plants are indicated with an asterisk (* *p* < 0.05 by Student’s *t*-test).

pH influences the solubility and ionic form of several elements in soil and affects nutrient availability for plant growth [41]. For instance, ferric ion precipitates as the utterly insoluble ferric hydroxide in an aerated solution with a pH greater than 8 [19]. Zn solubility reduces by 100-fold for each unit increase in pH [42]. Therefore, we speculated that high pH could affect the expression of *OsNAS*s even under Fe or Zn sufficiency. Then, we analyzed the expression levels of *OsNAS* genes in shoots and roots of the seedlings grown under high pH (pH 8.5) solution, along with or without Fe or Zn (Figure 3). Transcript levels of *OsNAS1* and *OsNAS2* were upregulated under alkaline growth solution, as compared to normal growth conditions (pH 5.5) (Figure 3a,b). Even in seedling roots supplemented with Fe and Zn, high pH could promote their expression, indicating that the bioavailability of Fe and Zn decreased due to the alkaline media. High pH without Fe led to the highest levels of *OsNAS1* and *OsNAS2* expression, respectively, relative to other growth conditions. However, the shoots and roots of the seedlings grown under both growth conditions showed significantly lower *OsNAS3* expressions in Fe-deficient media as compared to the seedlings cultivated with Fe (Figure 3c). Under Zn deficiency, no significant expression changes were observed for *OsNAS1* in seedling shoots and roots (Figure 3a). In seedling shoots and roots, *OsNAS2* expression increased upon Zn exclusion, but high pH raised it more (Figure 3b). Regardless of pH, *OsNAS3* rose in Zn-deficient seedlings (Figure 3c). According to these results, Fe or Zn deficiency combined with high pH causes significant expression changes in seedling shoots and roots, and *OsNAS* gene expression responded differently to high pH. Then, we investigated the growth response of WT, *2D*, *3D*, and *23D* seedlings under high pH Yoshida solution and a half-strength MS medium, with or without Fe or Zn (Figure 4 and Figure S3). In comparison to WT seedlings, all mutant seedlings of *2D*, *3D*, and *23D* displayed a considerable increase in shoot length when grown in a high-pH environment, irrespective of Fe or Zn supply. Significantly, the *23D* seedlings outperformed all others. For instance, in high-pH conditions, 2D, *3D*, and *23D* seedlings increased their shoot lengths by 25.9, 21.0, and 48.8% more than WT seedlings, respectively (Figure 4a,d and Figure S3a,d). At high-pH conditions without Fe, shoot lengths for *2D*, *3D*, and *23D* seedlings increased by 41.2%, 41.2%, and 75.6%, respectively, in comparison to WT seedlings (Figure 4b,d and Figure S3b,d). Moreover, in high-pH conditions without Zn, *2D*, *3D*, and *23D* seedlings’ shoot lengths increased by 21.1%, 20.0%, and 43.7%, respectively, in comparison to WT seedlings (Figure 4c,d and Figure S3c,d). Compared to WT, *2D*, and *3D*, our results suggest that the double-activation-tagged mutant *23D*, which simultaneously expresses higher levels of *OsNAS2* and *OsNAS3*, is better able to counteract the detrimental effects of high pH and high pH without Fe or Zn conditions on plant growth and development.

### 2.3. Increased Expression of Two OsNAS Genes Boosted Fe and Zn Contents in Mature Seeds

**Figure 5:**
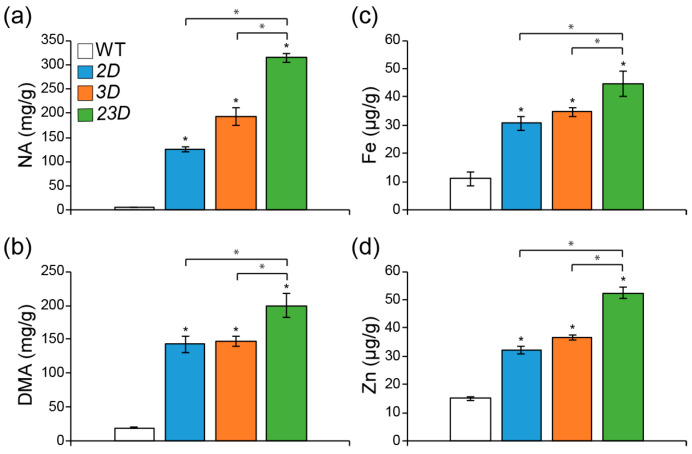
Increment of Fe and Zn concentrations in the mature seeds. Levels of NA (**a**), DMA (**b**), Fe (**c**), and Zn (**d**) in the unpolished seeds from WT and activation-tagged mutants. Data are means ± SE (n = 4), and significant differences among mutants and WT plants are indicated with an asterisk (* *p* < 0.05 by Student’s *t*-test).

To test if the double activation of *OsNAS2* and *OsNAS3* might further increase Fe and Zn concentrations, we measured Fe and Zn concentrations in the flag leaves of WT, *2D*, *3D*, and *23D* plants. We found that the mutant flag leaves had higher concentrations of Fe and Zn than the WT flag leaves (Figure S4). Importantly, flag leaves of *23D* contained the highest levels of Fe and Zn in comparison with other mutants. For instance, compared to the WT, Fe concentrations were 1.49-, 1.51-, and 1.87-fold elevated in the *2D*, *3D*, and *23D* mutants, respectively (Figure S4a), while Zn concentrations were 1.49-, 1.59-, and 1.94-fold elevated in the *2D*, *3D* and *23D* mutants, respectively (Figure S4b). Elevated amounts of Fe and Zn in flag leaves were correlated with increased amounts of NA and DMA in mature seeds, increasing the accumulation of Fe and Zn [18,19]. Therefore, we measured NA, DMA, Fe, and Zn concentrations in mature seeds (Figure 5). NA concentrations in mature seeds of *2D*, *3D,* and *23D* were found to be 20.2, 30.9, and 50.6 times higher, respectively, compared to WT (Figure 5a). In addition, DMA concentrations in *2D*, *3D* and *23D* seeds were also increased to being 7.2-, 7.4-, and 10.0-fold higher, respectively, compared to the WT (Figure 5b). Following the enhanced NA and DMA amounts, both Fe and Zn were observed to be enhanced (Figure 5c,d). Relative to mature WT seeds, Fe levels in *2D*, *3D,* and *23D* were 2.7, 3.1, and 4.0 times higher, respectively. (Figure 5c). We also performed Perls’ Prussian staining to visualize the accumulation and intensity of the Fe in the mature seeds and embryos (Figure S5). An enhanced blue color was observed in stained *2D*, *3D*, and *23D* mature seeds and embryos compared to the WT (Figure S5a,b). Notably, the strongest color observed visually was evident in mature *23D* seeds (Figure S5a) and embryos (Figure S5b), suggesting the highest accumulation of Fe. Furthermore, concentrations of Zn in mature *2D*, *3D,* and *23D* seeds were found to be 2.1, 2.4, and 3.5 times higher than those of WT, respectively (Figure 5d). These data suggest that dual activation of *OsNAS2* and *OsNAS3* could accumulate more Fe and Zn in rice grains than single-activation mutants of *OsNAS2* or *OsNAS3*.

**Figure 6:**
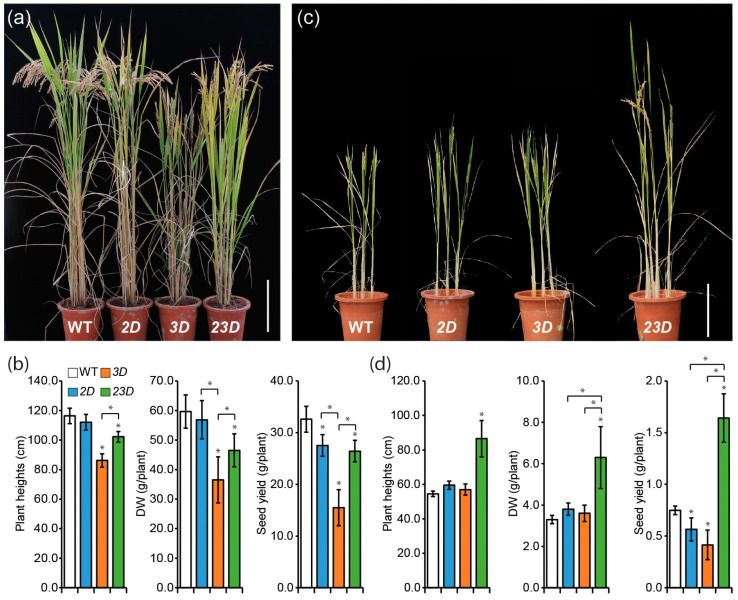
Representative plants of WT, *2D*, *3D,* and *23D*. (**a**) Comparison of WT, *2D*, *3D*, and *23D* plants grown in a paddy field. To take photographs, plants were transferred to pots from paddy fields before harvest. Scale bar = 20 cm. (**b**) Plant height, aboveground dry weight (DW), and seed yield of WT, *2D*, *3D*, and *23D* plants grown in a paddy field. Data are means ± SE (n = 4), and significant differences among mutants and WT plants are indicated with an asterisk (* *p* < 0.05 by Student’s *t*-test). (**c**) Morphological comparison of WT, *2D*, *3D*, and *23D* plants grown in alkaline soil (pH 8.0) at the reproductive stage. Scale bar = 20 cm. (**d**) Plant height, dry weight (DW), and seed yield of WT, *2D*, *3D*, and *23D* plants grown in alkaline soil (pH 8.0). Data are means ± SE (n = 4), and significant differences among mutants and WT plants are indicated with an asterisk (* *p* < 0.05 by Student’s *t*-test).

However, negative impacts on agronomic traits were observed for the matured mutant plants compared to WT plants when grown in paddy fields (Figure 6a,b). *2D* exhibited similar plant height and biomass as compared to the WT, but less seed yield (84.5% of WT) than the WT. Plant height, aboveground dry weight, and seed yield of *3D* were decreased to 74.1%, 61.2%, and 47.5% of WT, respectively. *23D*’s agronomic characteristics fell in between *2D* and *3D*. When the plants were grown in alkaline soil, the single activation-tagged mutants displayed similar plant height and biomass, but less seed yield as compared to WT (Figure 6c,d). Importantly, *23D* plants exhibited a highly significant increase in plant height, aboveground mass, and seed yield compared to all others (Figure 6c,d).

### 2.4. Increased Tolerance to Excess Metals during the Seedling Growth

**Figure 7:**
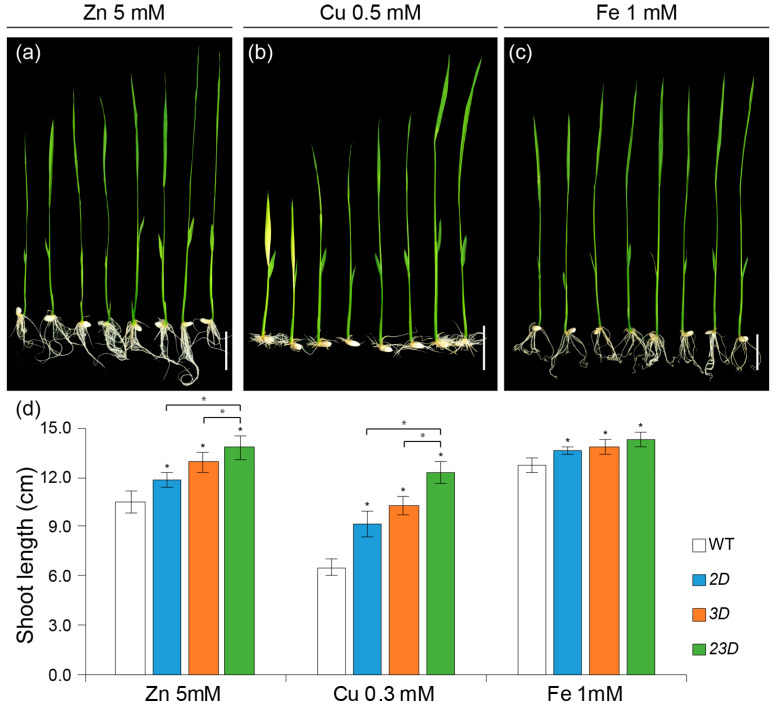
Growth test under the excess metal treatment. (**a**–**c**) Phenotypes of WT, *2D*, *3D*, and *23D* grown on half-strength MS solid containing 5 mM ZnSO_4_ (**a**), 0.5 mM CuSO_4_ (**b**), or 1 mM FeCl_3_ (**c**) for 10 days. Bars = 5 cm. Shoot lengths (**d**) of WT and activation-tagged mutants. Data are mean ± SE (n = 8), and significant differences among mutants and WT plants are indicated with an asterisk (* *p* < 0.05 by Student’s *t*-test).

NA is thought to participate in the detoxification of excess metals. Therefore, we verified the growth response of the mutants under excessive concentrations of various heavy metals (Figure 7 and Figure S6). *2D*, *3D*, and *23D* seedlings exhibited better growth under excessive amounts of Zn (5 mM), Cu (0.3 mM), Fe (1 mM), Ni (0.5 mM), and Co (0.5 mM) compared to the WT. Interestingly, the *23D* mutant seedling was the best performer under all the treatments of excess heavy metal concentrations (Figure 7 and Figure S6). For instance, shoot lengths in *2D*, *3D*, and *23D* were 12.4%, 22.9%, and 31.4% higher than the WT, respectively, at a higher Zn level (Figure 7a,d); shoot lengths of *2D*, *3D,* and *23D* were 40.6%, 57.8%, and 89.1% more than the WT, respectively, at an excess Cu level (Figure 7b,d); shoot lengths of *2D*, *3D,* and *23D* were 8.8%, 10.5%, and 13.8% more than the WT, respectively, at an excess Fe level (Figure 7c,d); shoot lengths of *2D*, *3D,* and *23D* were 21.5%, 30.0%, and 35.5% more than the WT, respectively, at an excess Ni level (Figure S6a,c); shoot lengths of *2D*, *3D,* and *23D* were 14.5%, 25.9%, and 42.5% more than the WT, respectively, at an excess Co level (Figure S6b,c). These data suggest that concomitant activation of *OsNAS2* and *OsNAS3* can improve plant tolerance at elevated heavy metal toxic conditions.

## 3. Discussion

Transgenic approaches, especially multiple gene expression, have already been proven effective to biofortify enhanced amounts of Fe and Zn in rice grains. Much research on the biofortification of Fe and Zn in rice grains has introduced one of the three *OsNAS* genes alone or in combination with other genes involved in metal homeostasis, but none of them have introduced two *OsNAS* genes at once. In this study, we generated a transgenic line (*23D*) with increased *OsNAS2* and *OsNAS3* expression to see if simultaneous activation of *OsNAS2* and *OsNAS3* would improve the accumulation of Fe and Zn in rice grains more than the single-activation-tagged mutants. Compared to the WT and single-activation-tagged mutants, NA and DMA levels were considerably higher in *23D*, and as a result, Fe and Zn levels were significantly raised in mature grains. In addition, *23D* had much higher concentrations of Fe and Zn in its leaves and roots, suggesting that NA and DMA had also accumulated there. Hence, the mutants were more resistant to Fe and Zn deficiency and high-pH conditions. In particular, *23D* plants outperformed all others by demonstrating more tolerance.

Combinatorial effects of *OsNAS2* and *OsNAS3* resulted in *23D*’s improved properties as compared to *2D* or *3D*. This might have been because *OsNAS2* and *OsNAS3* have differing physiological functions [15]. In other words, *OsNAS2* expression was present in Fe-sufficient root, and under Fe deficiency, its levels significantly increased in roots and leaves. *OsNAS3* expression was enhanced in roots but inhibited in leaves in response to Fe deprivation, although the *OsNAS3* transcript was present in both Fe-sufficient roots and leaves. In addition, Zn deficiency increased the expression of *OsNAS3* in roots and shoots while marginally decreasing the expression of *OsNAS2* in roots [31]. *OsNAS2* was found to be expressed in companion cells and pericycle cells close to the protoxylem in Fe-sufficient roots, according to promoter-GUS analysis. This suggests that *OsNAS2* has a role in DMA secretion for Fe absorption in roots. However, because *OsNAS3* is only expressed in the root pericycle and companion cells, it is possible that it is not involved in DMA secretion [15]. Moreover, *OsNAS3* expression was seen in the entire leaf tissues of the control plants [15]. Therefore, *OsNAS3* may work to produce NA or following parts such as DMA for the distribution of Fe and Zinc in rice plants.

NA is the precursor of DMA, which is synthesized by NAS, and NA is processed by NAAT and DMAS for synthesizing DMA [30,43]. We found that *OsNAAT1* and *OsDMAS1* were highly expressed in the mutants, with *23D* showing the highest expression. This may account for the increased levels of NA and DMA in *23D*. In rice, NA chelates Fe(II) and Zn(II) and carries them into the rice grains, and DMA chelates Fe(III) and transports it into root cells [2,13]. Through activating Fe(III)-DMA and Zn(II)-NA chelating processes and, likely, utilizing the *YSL* and Zn transporter genes, respectively, enhanced NA and DMA promote Fe and Zn accumulation in grains [26]. Moreover, mRNA levels of the Zn transporters (*OsZIP4* and *OsZIP5*), as well as the YSL transporters (*OsYSL2* and *OsYSL15*), were found to be much greater in mutants, which likely explains why transgenic plants and grains accumulate more Fe and Zn. The increased expression of the Fe transporter *OsIRT1* and the Fe storage protein *OsFer1* in *23D* may also be a contributing factor to the elevated levels of Fe and Zn. The mature grains, roots, and leaves of transgenic *OsIRT1* overexpressing plants were shown to have increased Fe and Zn concentrations [34].

However, the rice ferritins (*OsFer*) were not reported to be used for rice biofortification; rather, *OsFer* genes were found to be necessary for a positive response against Fe-induced oxidative stress [44]. Accordingly, *23D* seedlings were more tolerant to Fe toxic conditions, probably due to the elevated expression of *OsFer1* in *23D* than in *2D*, *3D*, and WT. Similarly, *OsNAS3* knockout plants were sensitive to excess Fe, whereas a transgenic rice line overexpressing barley *HvNAS1* with elevated NA production was tolerant of excess Fe [45]. While *23D* plants were the best performers under Zn, Cu, Ni, and Co toxicities, *2D* and *3D* plants also performed better under these conditions. This is likely because *23D* plants have improved abilities to chelate more of these metal ions than other plants, which reduces their toxic effects on plant growth.

Because of enhanced accumulation of Fe and Zn in shoots and roots due to higher levels of NA and DMA, transgenic plants were more resistant to Fe- and Zn-deficient conditions as well as high-pH conditions with or without Fe and/or Zn. As a result, *23D* plants would grow more successfully than single-activation mutants in alkaline growth conditions where high pH induces nutritional limitation. To maintain the solubility and availability of micronutrients to plants, soil pH is a crucial feature. Alkaline soils have reduced micronutrient availability, which has a detrimental effect on plant growth and yield [46]. For instance, Fe is abundant in soil but not easily soluble, especially in calcareous soils, which make up 30% of the earth’s surface [47]. In addition, the uptake of other metal micronutrients, such as Zn and Mn, can also be inhibited in alkaline soils [48]. The *23D* plants exhibited superior tolerance due to the combinatorial effects of *OsNAS2* and *OsNAS3*, as evident from the significant expression enhancement of *OsNAS2* and *OsNAS3* in the shoots and roots of rice seedlings under normal and high pH environments with or without Fe or Zn. Elevated expressions of *OsNAS* genes under high pH with or without Fe or Zn also explain the better tolerance of the mutants in alkaline conditions.

Although we measured Fe and Zn concentration only in the dehusked grains of the transgenic rice plants, we speculate that Fe and Zn amounts would be higher in the milled grains as well, with higher bioavailability. Intensified blue colors were observed for the whole transgenic grains and their embryos after Perls’ staining, where the strongest blue color in the *23D* grain was obvious. Furthermore, previous studies on the single-activation-tagged mutants, *2D* and *3D*, showed that the increased Fe and Zn in the milled grains were more bioavailable, as more NA and DMA would bind to more Fe and Zn and transport them into the grain endosperm [17,18,19]. In line with previous reports, *23D* seeds may accumulate more Fe and Zn bound to NA and/or DMA than *2D*, *3D,* or WT seeds, resulting in increased bioavailability. Mouse-feeding experiments can be used to test this in the future. An important point to consider while biofortifying rice is avoiding the negative impacts on normal plant growth and yield. Unfortunately, activation-tagged mutants displayed decreased growth and seed yield. Because they contained the higher amounts of Fe and Zn throughout all growth stages, they might have suffered from the oxidative stress, resulting to poor growth and reduction in yield. Although we did not observe any difference during the seedling stage, the influence increases over time and becomes more pronounced throughout the mature stage, making the negative effect more obvious. As a result, shorter plants with reduced yield were observed for *2D* and *3D* plants, probably for excess Fe-induced oxidative stress conditions in planta [19]. Similarly, *23D* plants showed almost resembling agronomic traits as observed in *2D*, but better than *3D*. However, when growth under high-pH soil, *23D* exhibited the greatest agronomic traits among WT and mutants, indicating the synergistic effect of OsNAS2 and OsNAS3.

Future studies on *23D* plants would benefit from their raised biofortified Fe and Zn levels as well as their increased tolerance to alkalinity and metal toxicity. By combining *OsNAS2* and *OsNAS3* with additional genes, future studies may further raise the Fe and Zn contents in rice grains.

## 4. Materials and Methods

### 4.1. Plant Materials and Growth

The Korean rice Dongjin (*Oryza sativa* L. ssp. *japonica*) was used as the experimental cultivar in this study. Two activation-tagged mutants (*OsNAS2-D1* and *OsNAS3-D1*) have been described previously [17,18]. Wild-type segregants from each progeny are referred to as wild-type (WT) progeny hereafter. Thirty-day-old seedlings of WT and activation-tagged mutant plants were transplanted into the paddy fields located at the Daegu Gyeongbuk Institute of Science and Technology (36° N) in 2020 under natural conditions. For the growth evaluation in calcareous soil, four-leaf-stage seedlings of WT and activation-tagged mutants were raised in pots (diameter 17 cm, height 18 cm) with nursery soil containing soluble CaCO_3_ to adjust to pH 8.0 under outdoor growth conditions until harvest. To prevent the soil from drying out, watering was performed frequently.

### 4.2. Hydroponic Culture for the Growth Test under Fe and Zn Deficiencies

The rice seeds were surface-sterilized, submerged in deionized water for 24 h in the dark, and then allowed to sprout for a further 24 h on wet filter paper. The uniform seedlings were selected and transferred into the containers containing Yoshida hydroponic solution for 15 days. Yoshida solution [49] with the following composition at full strength: NH_4_NO 1.43 mM, NaH_2_PO4·2H_2_O 0.32 mM, K_2_SO_4_ 0.51 mM, CaCl_2_·2H_2_O 1.00 mM, MgSO_4_·7H_2_O 1.64 mM, MnCl_2_·4H_2_O 9.47 μm, (NH_4_)_6_MO_7_O_24_·4H_2_O 0.07 μm, H_3_BO_3_ 18.88 μm, ZnSO_4_·7H_2_O 0.15 μm, CuSO_4_·5H_2_O 0.16 μm, FeCl_3_·6H_2_O 35.61 μm, citric acid (monohydrate) 70.83 μm. The pH of the working solution was adjusted to 5.5 with 1N sodium hydroxide (NaOH) every two days. To conduct our tests for micronutrient deficiencies, seedlings were moved into the Yoshida solution deficient in FeCl_3_·6H_2_O or ZnSO_4_·7H_2_O.

### 4.3. Seedling Growth on MS Solid Medium under Fe and Zn Deficiencies

WT and transgenic rice seeds were germinated on a standard MS agar medium supplemented with 0.1 100 μM Fe (III)-EDTA and 30 μM ZnSO_4_ as micronutrients [50]. Seedlings were grown for 10 days at 28 °C under continuous light. For our micronutrient deficiency tests, seeds were germinated and grown on an MS medium devoid of ZnSO4 or Fe (III)-EDTA for 10 days.

### 4.4. RNA Isolation and Quantitative Real-Time PCR

The total RNA was extracted from each tissue type with WelPrep total RNA isolation reagent (WELGENE, Gyeongsan, Republic of Korea), according to the manufacturer’s instructions, and treated with RNase-free DNase I (Takara Bio, Shiga, Japan) to remove the genomic DNA. First-strand cDNA was synthesized from 2 µg of total RNA in a 25 µL reaction mixture using the ImProm II Reverse Transcriptase system kit (Promega, Madison, WI, USA), followed by quantitative real-time PCR (qRT-PCR) analysis to determine gene expression levels (Bio-Rad, CFX96 Touch Real-Time PCR Detection System, USA) using a SYBR premix ExTaq kit (Takara Bio, Shiga, Japan). The rice *ubiquitin* gene (*LOC_Os06g46770*) was used to normalize the expression ratio for each gene, and changes in expression were calculated via the ΔΔ_Ct_ method. The gene-specific primers for PCR are listed in Table S1.

### 4.5. Measurement of Chlorophyll Concentrations

Chlorophyll was extracted from 0.1 g of leaf samples using 1 mL of 80% acetone [35]. After homogenization, the samples were incubated for 15 min on ice and spun for 10 min at 15,000× *g*, and then an aliquot of the supernatant fraction was collected for spectrophotometer measurements of *A*_663_ and *A*_643_. Chlorophyll concentrations, including chlorophyll *a* and *b*, were determined according to the method of [51].

### 4.6. Element Analysis in Plant Tissues

Samples from each tissue were dried at 70 °C until the weight remained constant, and then samples were digested in 1 mL of 11 N HNO_3_ for 3 days at 180 °C. After dilution, metal concentrations in the solutions were determined by inductively coupled plasma mass spectroscopy (ICP-MS, Varian 820-MS, Varian, Australia).

### 4.7. Perls’ Staining of the Mature Seeds

After the husk was manually removed, seeds were immersed in ultrapure water for four to five hours before being cut in two lengthwise through the embryo in a Petri dish using a razor blade. The cut seeds were submerged in freshly prepared Perls’ Prussian blue [52] solution (2% (*v*/*v*) HCl and 2% (*w*/*v*) potassium ferrocyanide), vacuum infiltrated for 15 min (500 mbar), and incubated at room temperature for 30 min. The seeds were then rinsed three times in distilled water and mounted in ultrapure water for visualization on a stereomicroscope.

### 4.8. Determination of NA and DMA Levels

Mature seeds were crushed into a fine powder with a Multi-Beads Shocker (Yasui Kikai, Osaka, Japan), and then 400 µL of 80% ethanol was added to the samples. The samples were centrifuged at 12,000× *g* for 5 min and supernatants were collected. Two more centrifugations were performed using the supernatants collected each time. Concentrations of NA and DMA were analyzed using the final supernatants by LC/ESI-TOF-MS, as previously described [53].

### 4.9. Excess Metal Tolerance

For the excess-metal treatments, seeds were germinated and grown for 10 days on solid media containing half-strength MS salts and supplemented with 5 mM ZnCl_2_, 0.3 mM CuCl_2_, 1 mM FeCl_3_, 0.5 mM NiCl_2_, or 0.5 mM CoCl_2_.

## Data Availability

Not applicable.

## Supplementary Materials

The following supporting information can be downloaded at https://www.mdpi.com/article/10.3390/ijms24076568/s1.
