# Supplementary material for: Concomitant Activation of OsNAS2 and OsNAS3 Contributes to the Enhanced Accumulation of Iron and Zinc in Rice
Source: Int J Mol Sci. 2023 Mar 31;24(7):6568. doi: 10.3390/ijms24076568 (PMC10095181; doi:10.3390/ijms24076568)
Supplement: Supplementary file 1 [file ijms-24-06568-s001.zip › ijms-2279226-supplementary.pdf]

Supplementary Data

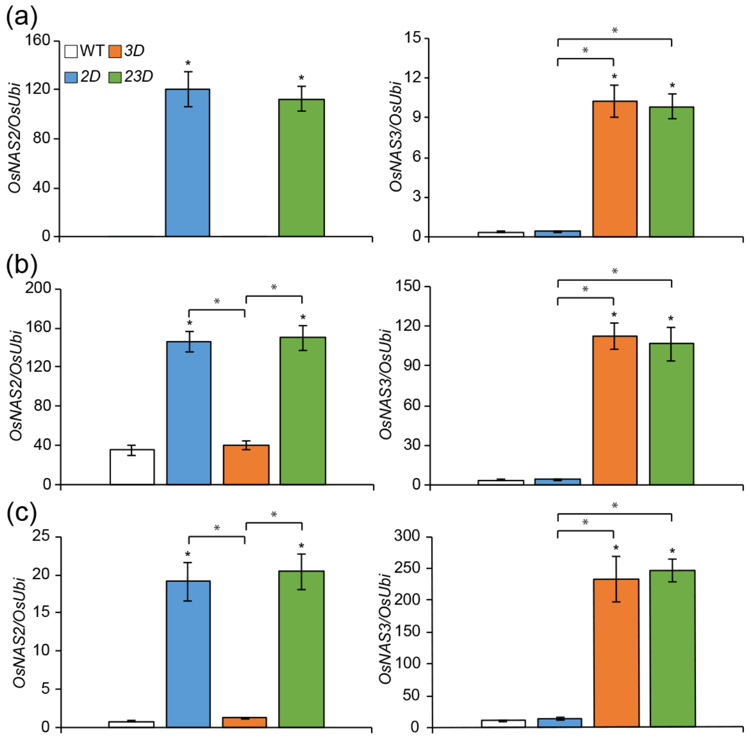

Figure S1. Real-time PCR analysis of WT and activation-tagged mutants. Expression levels of *OsNAS2* and *OsNAS3* were analyzed, using RNA from 7-day-old shoots (**a**) and roots (**b**) grown in ½ MS medium, and flag leaves (**c**) at 10 days after heading. Transcript levels are represented by ratios between *OsNAS2* or *OsNAS3* genes and the rice *ubiquitin* gene. Data are given as the mean ± SE (n = 3). WT indicates segregant wild-type plant; *2D, OsNAS2* activation-tagged mutant; *3D*, *OsNAS3* activation-tagged mutant; *23D*, the double activation-tagged mutant of *OsNAS2* and *OsNAS3*. Significant differences among WT and mutants plants are indicated with an asterisk (* $p < 0.05$ by Student's *t*-test).

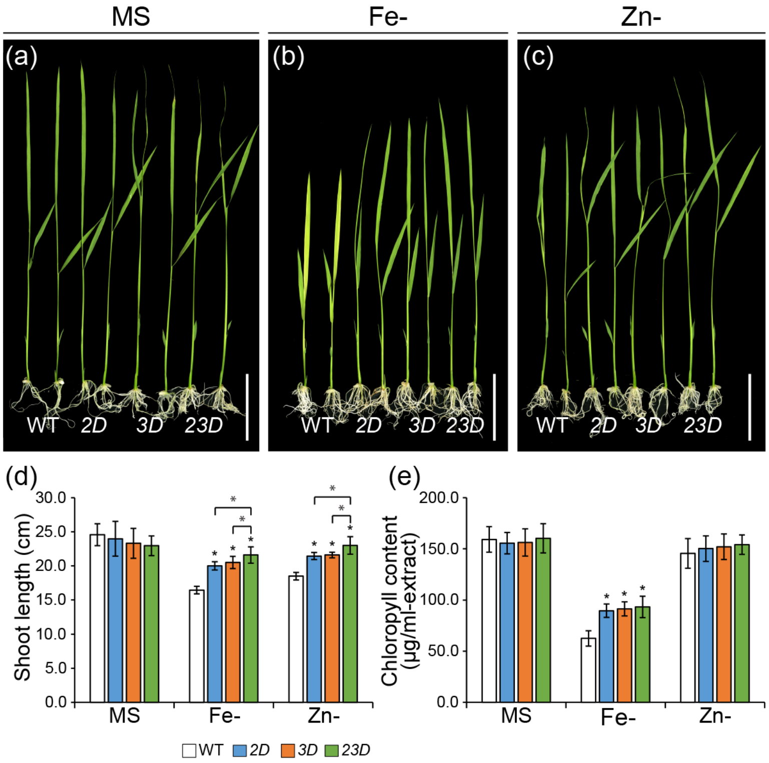

Figure S2. Growth of WT and activation-tagged mutants to Fe and Zn deficiencies. (**a**–**c**) Phenotypes of WT, *2D*, *3D*, and *23D* under control ½ strength MS solid medium (MS), or in Fe-deficient (Fe-) or Zn-deficient (Zn-) media for 15 days. Bars = 5 cm. Shoot lengths (**d**) and total chlorophyll concentrations (**e**) of WT and activation tagging mutants. WT indicates segregant wild-type plant; *2D, OsNAS2* activation-tagged mutant; *3D*, *OsNAS3* activation-tagged mutant; *23D*, double activation of *OsNAS2* and *OsNAS3*. Data are mean ± SE (n = 10 for plant height; n = 4 for chlorophyll contents). Significant differences among WT and mutants plants are indicated with an asterisk (* $p < 0.05$ by Student's *t*-test).

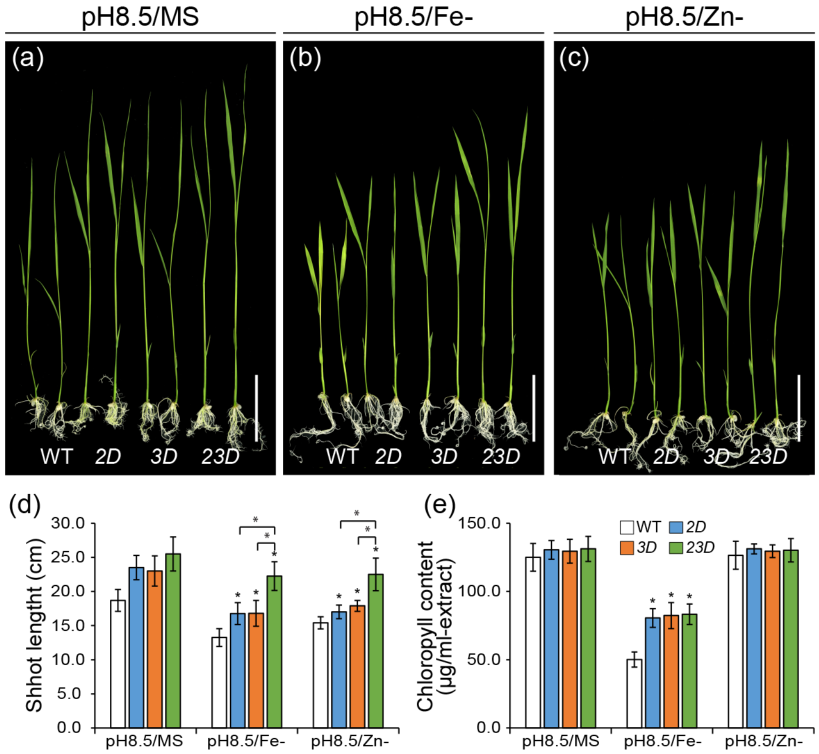

Figure S3. Growth test under high pH conditions. (**a**–**c**) Phenotypes of WT, *2D*, *3D*, and *23D* grown on alkaline half-strength MS solid medium solution (pH8.5), without Fe (pH8.5/Fe-), or without Zn (pH8.5/Zn-) for 15 days. Bars = 5 cm. Shoot lengths (**d**) and total chlorophyll concentrations (**e**) of WT and activation-tagged mutants. Data are mean ± SE (n = 8 for plant height; n = 4 for chlorophyll content) and significant differences between among WT and mutant plants are indicated with an asterisk (* $p < 0.05$ by Student's *t*-test).

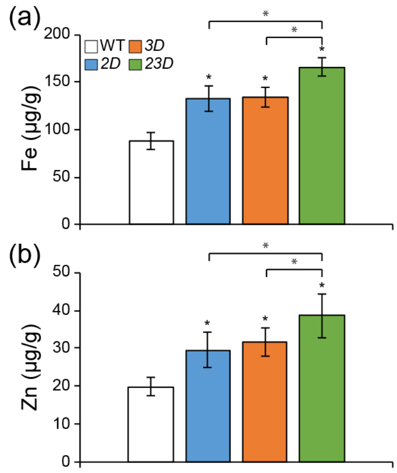

Figure S4. Measurement of Fe and Zn levels in flag leaves. Concentrations of Fe (**a**) and Zn (**b**) in flag leaves 10 days after heading. Error bars represent SE. Significant differences among WT and mutant plants are determined by Student's *t*-tests, $^{*}p < 0.05$.

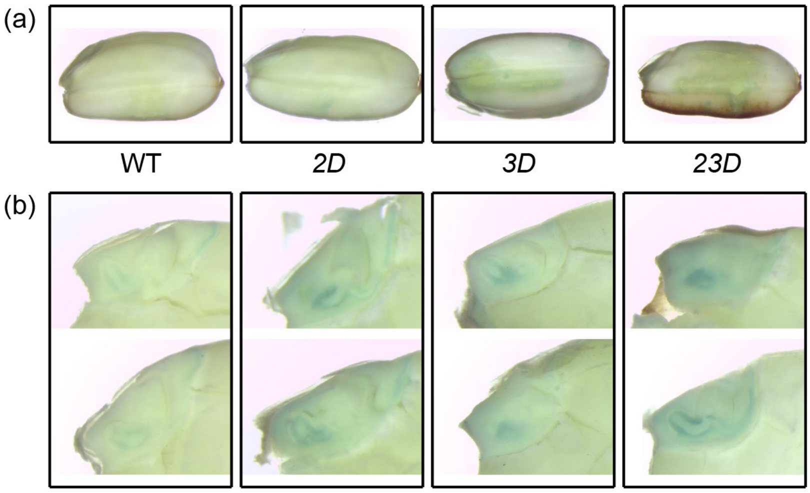

Figure S5. Fe distribution and intensity of the mature seeds. Perls' Prussian staining of unpolished grains (**a**) and embryos (**b**) from WT and activation tagging mutants. WT indicates segregant wild-type plant; *2D, OsNAS2*-activation tagging mutant; *3D*, *OsNAS3*-activation tagging mutant; *23D*, double-activation of *OsNAS2* and *OsNAS3*.

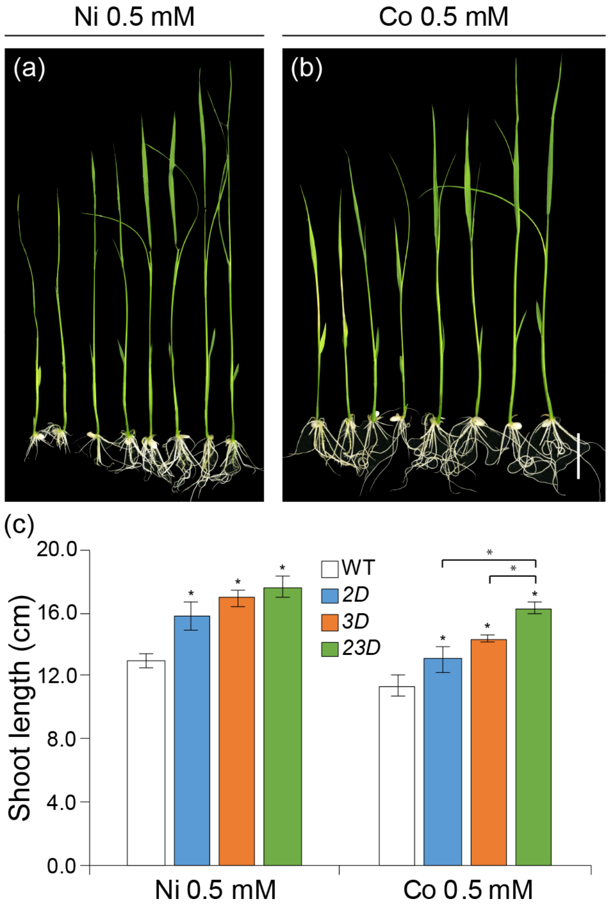

Figure S6. Growth test under the excess metal treatment. (**a**,**b**) Phenotypes of WT, *2D*, *3D*, and *23D* grown on ½ strength MS solid containing 0.5 mM $NiCl_2$ (**a**) or 0.5 mM $CoCl_2$ (b) for 10 days. Bars = 5 cm. (**c**) Shoot lengths of WT and activation-tagged mutants. Data are mean ± SE (n = 8) and significant differences among WT and mutant plants are indicated with an asterisk (* $p < 0.05$ by Student's *t*-test).

Table S1. Primers used in this study.

Primer	Sequence
OsNAS2-forward	5′-CGTCTGAGTGCGTGCATAGTA-3′
OsNAS2-reverse	5′-GAAGCACAAACACAAACCGATA-3′
OsNAS3-forward	5′-GTGATCAACTCCGTCATCATC-3′
OsNAS3-forward	5′-TCAGTCTCATCATGGGAAAAA-3′
OsUbi-forward	5'-CTGCTGCTGTTCTAGGGTTCAC-3'
OsUbi-reverse	5'-CAAAACGTTTCAGACACCATCA-3'
OsZIP4-forward	5′-GCGAAAGCAACAGTGATCATGGCGACTTTC-3′
OsZIP4-reverse	5′-GCAGCTCTTGGTTGCTCTGAAGATCTCATG-3′
OsZIP5-forward	5'-CTGGAGCTGGGAGTGGTGGT-3'
OsZIP5-reverse	5'-ATGTCGACGAGCGCCATGTA-3'
OsNAAT1-forward	5'-TAAGAGGATAATTGATTTGCTTAC-3'
OsNAAT1-reverse	5'-CTGATCATTCCAATCCTAGTACAAT-3'
OsDMAS1-forward	5'-GCCGGCATCCCGCAGCGGAAGATCA-3'
OsDMAS1-reverse	5'-CTCTCTCTCTCGCACCTGCTAGCGT-3'
OsIRT1-forward	5'-ATTCGCTGCATTGTTAGATTGA-3'
OsIRT1-reverse	5'-CATCATCAGTCACGAAGAACAAA-3'
OsOSYSL2-forward	5'-TCTGCTGGCTTCTTTGCATTTTCTG-3'
OsOSYSL2-reverse	5'-ACCATGTCGAACTCAGCATCCAGGA-3'
OsOSYSL15-forward	5'-GCGTTCGCCGTGCTCACGAACGTGG-3'
OsOSYSL15-reverse	5'-ATCCTCCACCCATGAAATTAAACAC-3'
OsFer1- forward	5'-TGGAAGCTGCGGGTATCCAT-3'
OsFer1-reverse	5'-TACTCAGCCTTGGCAATCCACA-3'
